# Draft Genome Sequence of Alkalihalobacillus clausii Strain AXA-BCL3, Isolated from the Sahastradhara Springs of Uttarakhand, India

**DOI:** 10.1128/mra.00926-22

**Published:** 2022-11-03

**Authors:** Amit Gaurav, Vishakha Bisht, Ruchir Gupta, Sanjay Shrivastav, Naveen Kumar Navani

**Affiliations:** a Department of Biosciences and Bioengineering, Indian Institute of Technology Roorkee, Roorkee, Uttarakhand, India; b AXA Parenterals Ltd., Roorkee, Uttarakhand, India; SIPBS, University of Strathclyde

## Abstract

Here, we report the draft genome sequence of Alkalihalobacillus clausii strain AXA-BCL3, which was isolated from a soil sample from the Sahastradhara springs of Uttarakhand, India. The genome was assembled in 125 contigs with a total length of 4,428,477 bp and a GC content of 44.5%. Genome annotation predicted 4,278 protein-coding genes and 75 tRNA genes.

## ANNOUNCEMENT

The use of probiotics to enhance human health has been practiced since ancient times. A significant number of studies have demonstrated the beneficial effects of probiotics for different health issues, like antibiotic-associated diarrhea. Alkalihalobacillus clausii is a potential probiotic strain that has been reported to have numerous health benefits, such as antimicrobial activity and immunomodulatory effects (1).

We isolated A. clausii strain AXA-BCL3 from soil samples collected from the Sahastradhara springs (30.39565° N, 78.12856° E) in the Dehradun district of Uttarakhand, India. Briefly, a 1-g soil sample (5- to 7-cm depth) was homogenized in 9 mL of normal saline (0.85% [wt/vol] NaCl) using a tabletop vortex (ThermoMixer C; Eppendorf, Germany) for 10 min. The homogenate (100 μl) was spread on brain heart infusion (BHI) agar. Plates were incubated at 37°C under normal atmospheric pressure for 24 h. Single isolated colonies were subjected to Gram staining. All Gram-positive rod-shaped isolates were subjected to identification using a microbial identification system (MicroStation ID system; BiOLOG, USA). One isolate was identified as *A. clausii*, which was further confirmed after 16S rRNA gene amplification and sequencing using 27F and 1492R universal primers (GenBank accession number OL799164.1, identified against the NCBI rRNA/ITS database; closest match, GenBank accession number NR_026140.1 [*A. clausii* strain DSM 8716 16S rRNA]) (2). Here, we report the genome sequence of *A. clausii* AXA-BCL3.

The total genomic DNA (gDNA) was extracted from a single isolated colony grown in BHI broth using a HiPurA bacterial genomic DNA purification kit (Himedia, India). The gDNA concentration was determined by the Qubit 3.0 fluorometer using a Qubit double-stranded DNA (dsDNA) HS assay kit (Thermo Fisher Scientific, USA). A sequence library was constructed using the NEBNext Ultra II DNA library prep kit for Illumina (New England Biolabs, USA). Both quantity and quality checks of the amplified library were performed in a Bioanalyzer 2100 instrument (Agilent Technologies) using a high-sensitivity DNA chip as per the manufacturer’s instructions. The Illumina HiSeq 4000 platform was used for sequencing the paired-end library in the 2 × 150-bp format. Sequencing yielded 5.6 million paired-end reads with a mean read length of 151 bp. Default parameters were used for all software unless otherwise specified. Quality trimming of Fastq sequences was performed using Trimmomatic (version 0.40) (3). Quality assessment was performed with FastQC (version 0.11.2) (http://www.bioinformatics.babraham.ac.uk/projects/fastqc) (4). *De novo* assembly of the bacterial genome was performed using Unicycler (version 0.4.8), which uses SPAdes as the *de novo* assembler (5). The genome was assembled in 125 contigs, with a total length of 4,428,477 bp, an *N*_50_ value of 81,185 bp, a coverage depth of 192×, and a GC content of 44.5%. Draft genome annotation was performed using the NCBI Prokaryotic Genome Annotation Pipeline (PGAP; version 6.1) (6), which identified 4,278 protein-coding sequences, 5 noncoding RNAs (ncRNAs), 75 tRNAs, 3 rRNA genes, and 62 pseudogenes. Analysis of secondary-metabolite biosynthesis gene clusters using BAGEL4 (version 1.2) revealed the presence of four ribosomally synthesized and posttranslationally modified peptide (RiPP) clusters, i.e., two clusters predicted to encode lantibiotics and sactipeptides (7). Analysis of antibiotic resistance determinants using ABRicate (https://github.com/tseemann/abricate) revealed the presence of two putative aminoglycoside resistance markers, i.e., *aadK* and *ant(4')-Ib*, one putative macrolide resistance marker, i.e., *erm(34)*, and one putative vancomycin resistance marker, *vanR*.

### Data availability.

The draft genome sequence of Alkalihalobacillus clausii strain AXA-BCL3 was deposited in the NCBI database under BioProject accession number PRJNA857844, BioSample accession number SAMN29638998, SRA accession number SRR20083698, and assembly number GCA_024608615.1.
